# Dietary yeast glycoprotein supplementation improves the growth performance, intestinal health and disease resistance of largemouth bass (*Micropterus salmoides*) fed low-fishmeal diets

**DOI:** 10.3389/fimmu.2023.1164087

**Published:** 2023-05-15

**Authors:** Wanjie Cai, Lele Fu, Haokun Liu, Jianhua Yi, Fan Yang, Luohai Hua, Linyue He, Dong Han, Xiaoming Zhu, Yunxia Yang, Junyan Jin, Jinjun Dai, Shouqi Xie

**Affiliations:** ^1^ State Key Laboratory of Fresh Water Ecology and Biotechnology, Institute of Hydrobiology, Chinese Academy of Sciences, Wuhan, China; ^2^ College of Advanced Agricultural Sciences, University of Chinese Academy of Sciences, Beijing, China; ^3^ The Hubei Provincial Key Laboratory of Yeast Function, Angel Yeast Co., Ltd, Yichang, China; ^4^ Hubei Engineering Research Center for Aquatic Animal Nutrition and Feed, Wuhan, Hubei, China; ^5^ The Innovative Academy of Seed Design, Chinese Academy of Sciences, Beijing, China

**Keywords:** yeast glycoprotein, largemouth bass, growth performance, intestinal mucosal morphology, intestinal immunity, intestinal antioxidation

## Abstract

The active ingredients extracted from yeast are important for regulating animal health. The aim of the current research was to explore the impacts of dietary yeast glycoprotein (YG) on the growth performance, intestinal morphology, antioxidant capacity, immunity and disease resistance of largemouth bass (*Micropterus salmoides*). A total of 375 juvenile fish (6.00 ± 0.03 g) were allocated into 15 fiberglass tanks. Triplicate tanks were assigned to each diet. The dietary YG inclusion was as follows: the first group was given a high fishmeal diet (40% fishmeal, 0% YG) (FM) and the second group was given a low fishmeal diet (30% fishmeal and 15% soybean meal, 0% YG) (LFM). The fish in the third, fourth and fifth groups were fed the LFM diet supplemented with 0.5% (LFM+YG0.5), 1.0% (LFM+YG1.0) and 2.0% (LFM+YG2.0) YG, respectively. After a 60- day feeding trial, a challenge test using *A. hydrophila* was carried out. The results showed that the final body weight (FBW) and weight gain rate (WGR) in the LFM+YG2.0 group were significantly higher than those in the LFM group and were no significantly different from those in the FM group. This may be partially related to the activation of the target of rapamycin (TOR) signaling pathway. Dietary YG supplementation enhanced intestinal physical barriers by upregulating the intestinal tight junction protein related genes (*claudin1*, *occludin* and *zo2*) and improving the structural integrity of the gut, which may be partially associated with AMPK signaling pathway. Moreover, dietary YG increased the antioxidant capacity in the gut, upregulated intestinal anti-inflammatory factors (*il-10*, *il1-1β* and *tgf-β*) and downregulated proinflammatory factors (*il-1β* and *il-8*), which may be partially related to the Nrf2/Keap1 signaling pathways. The results of the challenge test indicated that dietary supplementation with 0.5 or 1.0% YG can increase the disease tolerance of largemouth bass against *A. hydrophila*. In conclusion, the present results indicated that dietary supplementation with YG promotes the growth performance, intestinal immunity, physical barriers and antioxidant capacity of largemouth bass. In addition, 1.0% of dietary YG is recommended for largemouth bass based on the present results.

## 1 Introduction

In recent years, the aquaculture industry has grown rapidly and the demand for aquafeeds is increasing (1). Fishmeal is widely used as a high-quality protein source (high protein content, balanced amino acid composition and high palatability) in aquafeeds (2). However, the shortage of fishmeal resources and high prices make it unsustainable (3). The development of suitable protein sources to reduce the use of fishmeal is an urgent issue. Reducing the use of fishmeal in feed formulations will not only reduce costs and improve economic efficiency, but also promote the sustainable development of the aquaculture industry (4). Many potential novel protein sources have been investigated to reduce fishmeal content in fish feed, such as insect meal, bacterial proteins and plant proteins. Several potential alternative protein sources have been reported to be tested in many aquatic species (5–8). Plant proteins, such as soybean protein and cottonseed protein, have the advantages of being inexpensive and sustainable compared to fishmeal (9). Currently, plant protein is widely used as a substitute for fishmeal in aquafeeds. However, plant proteins contain various antinutritional factors, such as phytic acid, soya antigens and trypsin inhibitors, which have a series of negative impacts on cultured fish species, such as poor growth, deterioration of flesh quality and damage to the intestine (10–12). The use of feed additives seems to be an effective method to solve this problem. It was reported that supplementation with 0.25% creatine in soybean-substituted fishmeal feed promoted the growth performance and enhanced the meat quality of spotted seabass (*Lateolabrax maculatus*) (13). Taurine supplementation increases the replacement level of fishmeal by soybean concentrate in diets of pacific white shrimp (*Litopenaeus vannamei*) (14).

As functional feed additives, yeast products are widely applied in aquaculture to improve the growth and health of cultured fish (15). The yeast glycoprotein (YG) studied here is a substance with antibacterial activity that is released after directed enzymatic hydrolysis of yeast cells; it contains mainly glycoprotein, glucan and mannan oligosaccharide (MOS). A study reported that glucan alleviated hepatic dysfunction and resistance to *Aeromonas sobria* caused by atrazine in Nile tilapia, which indicates that glucan can improve the immunity of fish and reduce resistance to bacterial infections (16). MOS is another important immunomodulatory compound. It was reported that MOS improved the survival rate of Pacific white shrimp (*Litopenaeus vannamei*) under exposure to *Vibrio harveyi* (17). The above results confirmed the potential of YG in promoting growth and enhancing the immunity of organisms. Dietary YG has been reported to ameliorate growth performance and improve intestinal morphology and the immune response in piglets (18). Nevertheless, few studies have explored the impacts of YG on aquatic animals.

Numerous studies have shown that the intestine is the main site of nutrient digestion and absorption (19). It also has a certain immune function, playing key roles in maintaining fish health (20). Intestinal immune substances are involved in immune responses that prevent and combat invasion by pathogenic bacteria (21). The inflammatory response mediated by cytokines is considered a vital component of the immune response. Anti-inflammatory factors and proinflammatory factors are present in the fish gut. Intestinal structural integrity is also important for maintaining intestinal health. The composition of the tight junction complex and the antioxidant capacity are important to intestinal structural integrity (22). Notably, YG contains longer unfolded glycoproteins that provide more sites to interact with immune cells in the host gut. Therefore, there is a great need to explore the impacts of YG on the intestinal immunity and health of farmed fish.

Largemouth bass (*Micropterus salmoides*) is an important carnivorous fish that has been widely cultivated due to its delicious flesh, rapid growth and strong adaptability (23). However, high densities and intensive farming patterns result in oxidative stress and infectious diseases occurring frequently in largemouth bass farming. Thus, the purpose of this study was to investigate the impacts of YG on the growth performance, intestinal immunity, antioxidant capacity and disease resistance of largemouth bass. This study may contribute to assessing the potential of yeast products as an alternative to antibiotics.

## 2 Materials and methods

The experimental animals were cared for and slaughtered based on the Guidance of the Care and Use of Laboratory Animals. The experimental subject was approved by the Institute of Hydrobiology, Chinese Academy of Sciences (IHB, CAS, Protocol No. 2016-018).

### 2.1 Experimental diets

In this experiment, five (crude protein, 50%; crude lipid, 5%) experimental diets were formulated and the approximate composition is presented in **Table 1**. The nutritional components of yeast glycoprotein are showed in **Table 2**. The first diet contained 40% fishmeal and 18.5% casein, which was a high fishmeal diet for this experiment (FM). The second diet contained 30% fishmeal, 15% soybean meal and 18.5% casein, which was a low fishmeal diet (LFM). Third, fourth and fifth were contained same protein source as LFM and were supplemented with 0.5% (LFM+YG0.5), 1.0% (LFM+YG1.0) and 2.0% YG (LFM+YG2.0). The YG in present study was provided by Angel Yeast Co., Ltd (Hubei, China). The process of feed production refers to previous work in our laboratory (19). An oven was used to dry all diets at 60 °C and then stored in a refrigerator.

**Table 1:** Formulation and compositions of experimental diets (% dry matter).

Ingredients(%)	FM	LFM	LFM+YG0.5	LFM+YG1.0	LFM+YG2.0
Fishmeal^1^	40.00	30.00	30.00	30.00	30.00
Soybean meal	00.00	15.00	15.00	15.00	15.00
Casein	18.50	18.50	18.50	18.50	18.50
Wheat protein concentrate	4.20	3.50	3.50	3.50	3.50
Wheat flour	10.00	10.00	10.00	10.00	10.00
Tapioca	11.90	11.90	11.90	11.90	11.90
Yeast glycoprotein^2^	0.00	0.00	0.50	1.00	2.00
Fish oil	4.00	4.54	4.54	4.54	4.54
Vitamin and mineral premix^3^	2.00	2.00	2.00	2.00	2.00
Monocalcium phosphate	1.50	1.50	1.50	1.50	1.50
Choline chloride^4^	0.20	0.20	0.20	0.20	0.20
Methionine	0.00	0.15	0.15	0.15	0.15
Cellulose	7.70	27.06	26.56	26.06	25.06
Approximate composition						
Moisture (%)	7.24	7.07	7.84	7.54	7.50
Crude protein (%)	50.32	49.92	48.96	48.88	50.39
Crude lipid (%)	5.33	5.37	5.64	5.30	5.08
Ash (%)	9.02	9.05	9.64	8.38	8.80
Methionine (%)	1.02	1.02	1.09	1.01	1.04
Gross energy (kJ/g)	18.41	18.43	18.24	18.24	18.45

^1^ Fishmeal was purchased from Coland Feed Industry (Wuhan, Hubei, China).

^2^ Yeast glycoprotein was purchased from ANGEL YEAST Co. Ltd. (Yichang, Hubei, China).

^3^ The vitamin and mineral premix was formulated following Gong et al. (23).

^4^ Choline chloride was composed of 50% choline chloride and 50% silicon dioxide.

**Table 2:** The nutritional components in yeast glycoprotein (%) ^a^.

Main components	%
Moisture	5.40
Crude protein	17.50
Mannan oligosaccharide (MOS)	22.60
β- glucan	21.40

a Data from ANGEL YEAST Co. Ltd. (Yichang, Hubei, China).

### 2.2 Fish and feeding trial

The experimental fish were obtained from a freshwater fish farm (Hubei, China). After 3 weeks of acclimation, 375 fish with similar sizes (6.00 ± 0.03 g) were divided into 15 fiberglass tank (150 L) with 25 fish per tank. The diets were allocated to triplicate tanks. During the 60-days feeding period, experimental fish were hand-fed daily at 8:30 and 16:30 until apparent satiety. The water temperature in tank was measured every day and which was kept at 28.0 ± 1.2 °C. The dissolved oxygen > 6.0 mg/L, pH was maintained at 6.8–7.2 and the concentration of ammonia nitrogen < 0.5 mg/L.

### 2.3 Bacterial challenge

The bacteria in bacterial challenge and the culture procedure refer to previous study in our lab (23). A pre-experiment was carried out to confirm the 5-day LC50 (The concentration of bacteria could result in 50% of fish mortality) before challenge test. The result of pre-experiment suggested that the 5-day LC50 was 1×10^8^ CFU/mL. After 60-days feeding period, 16 fish per tank were injected intraperitoneally with 1 × 10^8^ CFU/mL *A. hydrophila*. After bacteria challenge, fish mortality of experimental fish was recorded continuously for 5 days.

### 2.4 Sample collection

At the end of the feeding period, the weight and total number of all fish in each tank were weighted and counted. Two experimental fish per tank were selected and anesthetized with MS-222 (50 mg/L) for 5 min. The blood, liver, middle and hindgut were sampled. Blood was quickly collected from dorsal vessel of fish by syringes and centrifuged (3500 g for 10 min). The obtained plasma sample was stored at - 80°C for subsequent analysis. The liver and gut were quickly collected at ice and stored at - 80°C.

### 2.5 Biochemical assays

The chemical compositions (moisture, crude protein, crude lipid and ash) of feeds and fish samples were performed following the methods of AOAC (24). The detailed procedure refers to previous work (25). The levels of complement 3 (C3, H186-1-2), complement 4 (C4, H184-2-2) and immunoglobulin M (IgM, H109-1-2) in middle gut were measured by Elisa kits. The kits are pre-coated with antibodies that bind specifically to these substances in fish tissues. The activities of acid phosphatase (ACP) (A060-1-1) and alkaline phosphatase (AKP) (A059-2), including lysozymes (LZM) (A050-1-1), Total antioxidant capacity (T-AOC, A015-2-1), superoxide dismutase (SOD, A001-3-2) and catalase (CAT, A007-1-1) of middle intestine were tested using colorimetric methods. These kits provide substrates that can only be reacted with corresponding enzymes. All kits were purchased from Nanjing Jiancheng Bioengineering Institute (Jiangsu, China).The total triglyceride (TG), total cholesterol (TC), high-density lipoprotein cholesterol (HDL-C), low-density lipoprotein cholesterol (LDL-C), aspartate aminotransferase (AST) alanine aminotransferase (ALT) and alkaline phosphatase (ALP) contents in plasma were measured by an automatic biochemical analyzer (Mindray BS-460, Shenzhen, China) (TG (P/N:105-000449-00), TC (P/N:105-000448-00), HDL-C (P/N:105-000463-00), LDL-C (P/N:105-000464-00), AST (P/N:105-000443-00), ALT (P/N:105-000442-00) and ALP (P/N:105-000444-00)).

### 2.6 Histological analysis

To observe the impacts of YG on the middle intestinal structure of largemouth bass. The H&E staining was performed in intestine paraffin sections by Servicebio Company (Wuhan, China). a fully automatic digital slide scanner (Aperio VERSA 8, Leica, Germany) was used to obtain the intestine images from each slide. The image was analyzed by ImageJ Launcher software.

### 2.7 Gene expression and western blot analysis

The total RNA extraction of liver and middle intestine refers to previous study in our lab (25). The information of primers used for quantitative RT–PCR were showed in **Table 3**. The method of Vandesompele was applied to calculate the results (27).

**Table 3:** Primers used for quantitative RT-PCR (qPCR).

Accession no.	Gene name	Primer sequence (5’-3’)	Annealing temp. (°C)	Product length(bp)
XM_038723321.1	*tor* ^1^	F: TCAGGACCTCTTCTCATTGGC R: CCTCTCCCACCATGTTTCTCT	60	208
XM_038713349.1	*s6* ^2^	F: GCCAATCTCAGCGTTCTCAAC R: CTGCCTAACATCATCCTCCTT	60	156
XM_038729709.1	*eif4e^3^*	F: TACCAAAAGCGGTTCAACCAC R: GCAGCACTCTAACTCTCATCC	60	283
XM_046070501.1	*4ebp^4^*	F: TGGACATCTAACAGACTGGA R: GCCACACTGTACCTAGATGT	60	196
XM_038718401.1	*claudin1*	F: GATCAGAGCCACTACCCCAA R: TTCCAAAGCCCTTCATACAGC	58	279
XM_038694323.1	*claudin4*	F: ATGTACTCTGCAGGAGTGGA R: AGCATGGAGTCGTGCACTCT	60	212
XM_038715419.1	*occludin*	F: CAGCCCTTCAGAGGAGAC R: CTACAGCCTGGTATTTGG	58	335
XM_038701018.1	*zo1* ^5^	F: AATACACTCTCCCCAAAACGG R: GCGAAGACCACGAAATCTCC	58	65
XM_038733200.1	*zo2* ^6^	F: GTCGTACCGCTCCTACTC R: TTCTTGGTCCTCTATGCTC	58	301
XM_046060334.1	*acp^7^*	F: TATGAAGAAACATGACGTGCC R: TCAAAGTCCTCGTCACTCCC	60	231
XM_038696722.1	*akp^8^*	F: TTGGACCGGAAGCTTAACACC R: TCAAAGTCCTCGTCACTCCC	60	201
XM_038704976.1	*cat^9^*	F: GTTCCAGCTATCTTTTAACCC R: AAGAGAGGCACATAAATGCAA	60	75
XM_038708943.1	*sod^10^*	F: TCCCCACAACAAGAATCATGC R: TCATCAGCCTTCTCGTGGA	53	180
XM_038713810.1	*lzm* ^11^	F: TCCAATGATGTTGTTGCCAGA R: AAGCCATTGATTTTGTACCAC	58	47
XM_046034892.1	*il1β* ^12^	F: CGTGACTGACAGCAAAAAGAGG R: GATGCCCAGAGCCACAGTTC	54	166
MW751832.1	*il8* ^13^	F: CGTTGAACAGACTGGGAGAGATG R: AGTGGGATGGCTTCATTATCTTGT	60	112
XM_038710731.1	*tnfα* ^14^	F: CTTCGTCTACAGCCAGGCATCG R: TTTGGCACACCGACCTCACC	60	161
XM_038696252.1	*il10^15^*	F: CGGCACAGAAATCCCAGAGC R: CAGCAGGCTCACAAAATAAACATCT	60	119
Yu et al., 2019 (26)	*il11β* ^16^	F: TTCCCAACAGACAGATGAAGAACTC R: TGCCTGTGTTCAGCCAGTCAA	60	182
XM_038693206.1	*tgfβ* ^17^	F: GCTCAAAGAGAGCGAGGATG R: TCCTCTACCATTCGCAATCC	58	118
XM_038734014.1	*ampkα* ^18^	F: AGGCGAGCTCTTCGACTACA R: CCAAAGTCTG CAATCTTGGC	60	183
XM_038709736.1	*acc* ^19^	F: ATCCCTCTTTGCCACTGTTG R: GAGGTGATGTTGCTCGCATA	60	121
XM_038720536.1	*nrf2* ^20^	F: TCCCCAGAGCAGACAGTTCC R: CTCCATTTGCATGTTCAGGC	58	162
XM_038728592.1	*keap1* ^21^	F: GCACCTAACCGTGGAACTCAA R: CCAGTTTTAGCCAGTCATTGTTCC	58	109
XM_038695351.1	*β-actin*	F: AAAGGGAAATCGTGCGTGAC R: AAGGAAGGCTGGAAGAGGG	60	184

^1^tor, Target of rapamycin; ^2^s6, Ribosomal protein S6 kinase 1; ^3^eif4e, Eukaryotic translation initiation factor 4E; ^4^4ebp, Eukaryotic translation factor 4E-binding protein; ^5^zo1, Tight junction protein 1; ^6^zo2, Tight junction protein 2; ^7^acp, Acid phosphatase; ^8^akp, Alkaline phosphatase; ^9^cat, Catalase; ^10^sod, Superoxide dismutase [Cu-Zn]; ^11^lzm, Lysozyme -like transcript; ^12^il1β, Interleukin-1β; ^13^il8, Interleukin 8; ^14^tnfα, Tumour necrosis factor α; ^15^il10, Interleukin-10; ^16^il11β, Interleukin-11β; ^17^tgfβ, Transforming growth factor β; ^18^ampkα, 5’-AMP-activated protein kinase catalytic subunit α; ^19^acc, Acetyl-CoA carboxylase; ^20^nrf2, Nuclear factor erythroid 2-related factor 2a; ^21^keap1, Kelch-like ECH-associated protein 1.

The method and detailed procedure of liver western blot refers to previous study in our lab (25). The primary antibodies containing P-TOR (#2971; CST, Danvers, MA, United States), TOR (#2972; CST, Danvers, MA, United States), P-S6 Ser235/236 (#4858; CST, Danvers, MA, United States), S6 (#2217; CST, Danvers, MA, United States). The density of membranes with proteins complexes were quantified by Image J software (National Institutes of Health). GAPDH was used as an internal reference protein.

### 2.8 Statistical analysis

All data are expressed as mean ± standard error (SE), which were statistically analyzed by SPSS 20.0 software (IBM, USA) and performed to one-way analysis of variance (ANOVA). The significance of differences between treatments was detected by Duncan’s multiple range test. Differences were regarded as significant at *P* < 0.05.

## 3 Results

### 3.1 Growth performance, nutritional compositions of the whole body and cumulative survival rate after challenge

The growth performance is presented in **Table 4**. The final body weight (FBW) and weight gain rate (WGR) in the treatments with YG supplementation were higher than those in LFM group. Especially, those in the LFM+YG2.0 group were significantly higher than those in the LFM group (*P* < 0.05), and those in LFM+YG2.0 group were no significant difference compared with those in the FM group. The feed efficiency (FE) was no significant difference compared to that in LFM group, but significantly lower than that in FM group (*P* < 0.05). No significant difference was found in the feeding rate (FR) or survival rate (SR) among the groups. The whole-body nutritional compositions are also shown in **Table 4**. The crude lipid content in LFM+YG0.5 group was significantly higher than that in the LFM group (*P* < 0.05). No significant difference in lipid content between the treatments with YG supplementation and the FM group. In addition, there is no remarkable different in contents of the whole-body moisture, crude protein and ash of all groups.

**Table 4:** Effects of dietary supplementation with YG on the growth performance and whole-body composition of largemouth bass.

Parameters	FM	LFM	LFM+YG0.5	LFM+YG1.0	LFM+YG2.0	*P* value
Growth performance						
IBW g^1^	6.03 ± 0.01	5.98 ± 0.00	6.01 ± 0.01	5.99 ± 0.01	6.02 ± 0.01	0.062
FBW g^2^	44.61 ± 0.50^c^	39.30 ± 0.32^a^	41.22 ± 0.63^ab^	41.33 ± 0.32^ab^	42.80 ± 1.38^bc^	0.015
WGR %^3^	639.50 ± 9.24^c^	557.53 ± 5.65^a^	586.10 ± 10.90^ab^	590.53 ± 5.30^ab^	610.47 ± 23.13^bc^	0.026
FR %BW/d^4^	2.06 ± 0.05	2.13 ± 0.02	2.17 ± 0.02	2.14 ± 0.01	2.14 ± 0.01	0.124
FE %^5^	132.26 ± 2.65^b^	123.29 ± 1.69^a^	122.88 ± 1.93^a^	124.87 ± 0.58^a^	125.86 ± 1.12^a^	0.028
SR %^6^	98.00 ± 0.02	100.00 ± 0.00	98.68 ± 0.01	100.00 ± 0.00	100.00 ± 0.00	0.431
Whole-body composition						
Moisture %	74.06 ± 0.28	75.43 ± 0.34	75.06 ± 1.20	74.75 ± 0.51	74.53 ± 0.30	0.721
Crude protein %	16.67 ± 0.41	16.04 ± 0.10	15.74 ± 0.72	16.32 ± 0.28	16.45 ± 0.22	0.594
Crude lipid %	4.14 ± 0.18^ab^	3.78 ± 0.20^a^	4.76 ± 0.26^b^	4.35 ± 0.12^ab^	4.28 ± 0.06^ab^	0.036
Ash %	3.46 ± 0.06	3.26 ± 0.10	3.17 ± 0.18	3.28 ± 0.03	3.39 ± 0.02	0.229

Data are presented as the Means ± SE (n = 3). Values within the same row with different letters are significantly different (P < 0.05).

^1^IBW, initial body weight (g).

^2^FBW, final body weight (g).

^3^WGR, weight gain rate (g) = 100 × (final mean weight − initial mean weight)/initial mean weight.

^4^FR, feeding rate (% body weight day ^−1^) = 100 × (feed intake in dry matter)/[days × (initial body weight+ final body weight)/2].

^5^FE, feed efficiency (%) = (final body weight-initial body weight)/feed intake in dry matter.

^6^SR, survival rate (%) = 100 × (final fish number/initial fish number).

**Figure 1:**
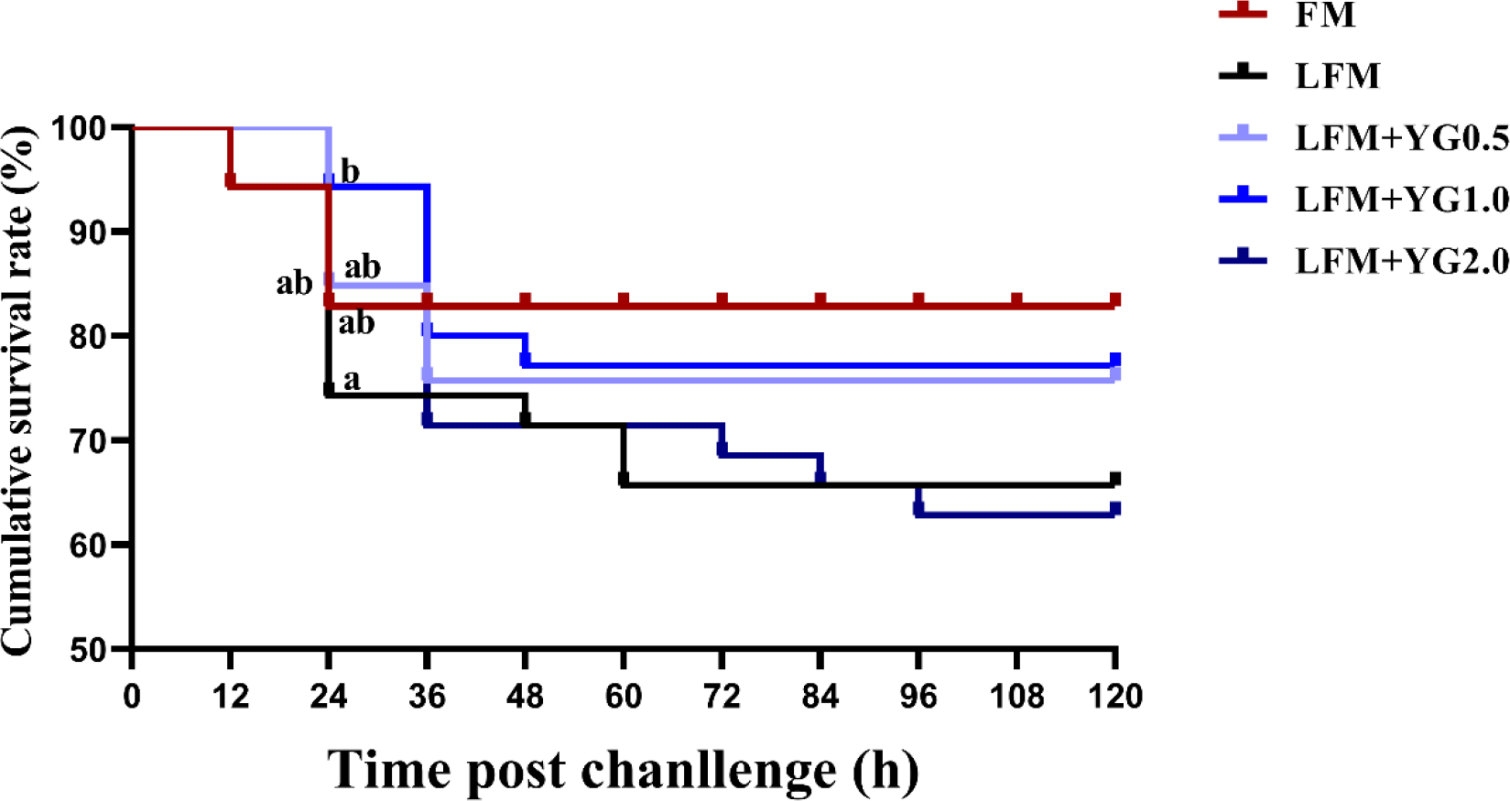
Effects of dietary supplementation with YG on postchallenge survival of largemouth bass after infection with *A. hydrophila*. The cumulative survival rate of the largemouth bass was recorded and analysed. The equation is expressed as survival rate (%) = (final number of fish survivor/initial number of inoculated fish) × 100.

The results showed that the 24-hour cumulative survival rates of the largemouth bass were increased in the YG-supplemented groups compared with the LFM group, and a significant increase was observed in the LFM+YG1.0 group (**Figure 1**) (*P* < 0.05). Moreover, there was no significant difference between the FM and experimental groups. The 120-hour cumulative survival rate in the FM group was 82.58 ± 0.76%, which was the highest among all groups. This was followed by 77.78 ± 18.22% in the LFM+YG1.0 group, 72.73 ± 5.25% in the LFM+YG0.5 group, 66.16 ± 9.19% in the LFM group and 63.38 ± 0.76% in the LFM+YG2.0 group.

### 3.2 Hematological parameters

The hematological parameters of the largemouth bass are presented in **Table 5**. No remarkable difference was found in plasma TG levels in all groups. Furthermore, that in LFM+YG1.0 and LFM+YG2.0 groups was remarkably decreased compared with the LFM treatment (*P* < 0.05). The levels of plasma HDL-C and LDL-C showed no remarkable difference among all groups. In addition, the plasma AST and ALP levels in LFM+YG1.0 group were remarkably decreased than those in the LFM group (*P* < 0.05). plasma ALT showed no significant difference in all treatments.

**Table 5:** Effects of dietary supplementation with YG on the hematological parameters of largemouth bass.

Parameters	FM	LFM	LFM+YG0.5	LFM+YG1.0	LFM+YG2.0	*P* value
TG mmol/L^1^	4.15 ± 0.50	3.72 ± 0.36	3.68 ± 0.37	4.58 ± 0.14	4.04 ± 0.35	0.343
TC mmol/L^2^	7.20 ± 0.15^a^	9.30 ± 0.41^c^	8.63 ± 0.25^bc^	7.88 ± 0.29^ab^	7.37 ± 0.25^a^	0.001
HDL-C mmol/L^3^	3.28 ± 0.09	3.02 ± 0.16	2.73 ± 0.31	3.11 ± 0.05	3.06 ± 0.13	0.379
LDL-C mmol/L^4^	1.67 ± 0.05	1.64 ± 0.11	1.47 ± 0.16	1.76 ± 0.05	1.60 ± 0.16	0.582
ALT U/L^5^	13.75 ± 1.42	18.00 ± 0.51	16.05 ± 2.49	16.13 ± 1.68	18.67 ± 1.02	0.318
AST U/L^6^	80.60 ± 6.48^a^	112.20 ± 3.55^c^	100.72 ± 6.15^bc^	91.27 ± 6.09^ab^	101.40 ± 5.76^bc^	0.015
ALP U/L^7^	129.05 ± 3.51^a^	177.08 ± 4.43^c^	166.48 ± 2.29^bc^	148.03 ± 10.92^ab^	160.48 ± 9.40^bc^	0.003

Data are presented as the Means ± SE (n = 6). Values within the same row with different letters are significantly different (P < 0.05).

^1^TG, Total triglyceride (mmol/L).

^2^TC, Total cholesterol (mmol/L).

^3^HDL-C, High-density lipoprotein cholesterol (mmol/L).

^4^LDL-C, Low-density lipoprotein cholesterol (mmol/L).

^5^ALT, alanine aminotransferase (U/L).

^6^AST, aspartate aminotransferase (U/L).

^7^ALP, alkaline phosphatase (U/L).

### 3.3 Expression of key proteins and related genes in protein synthesis

**Figure 2:**
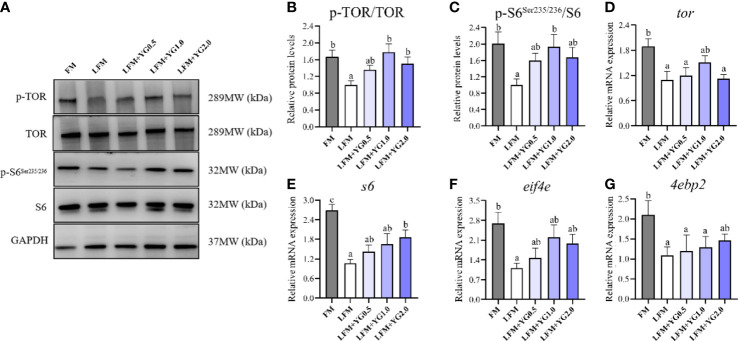
Effects of dietary supplementation with YG on protein synthesis metabolism in the liver of largemouth bass. **(A)** Protein and phosphorylation levels of TOR and s6 in the liver. **(B)** Quantification of TOR protein phosphorylation. **(C)** Image quantification results of s6 protein phosphorylation. **(D)** Relative mRNA expression of *tor*. **(E)** Relative mRNA expression of *s6*. **(F)** Relative mRNA expression of *eif4e*. **(G)** Relative mRNA expression of *4ebp2*. Columns represent the mean ± SEM (n = 6). For each index, bars not sharing a common letter indicate significant differences (*P* < 0.05).

Expression of key proteins and related genes of protein synthesis in the liver of the largemouth bass are presented in **Figure 2**. Compared with the LFM group, the protein phosphorylation level of the TOR in the LFM+YG1.0 and LFM+YG2.0 groups was significantly increased (*P* < 0.05) (**Figures 2A, B**). Nevertheless, No significant difference between the LFM+YG0.5, LFM+YG1.0, LFM+YG2.0 and FM groups. Accordingly, the transcriptional expression of *tor* in LFM+YG1.0 groups was no significant difference compared to that in the FM group (**Figure 2D**). Similar results were observed for the expression of s6 at protein and transcriptional levels. As shown in **Figures 2A, C, E**, the protein phosphorylation level of s6 in the LFM+YG1.0 group was significantly increased than LFM group (*P* < 0.05). With the increase in dietary YG levels, the transcriptional expression of *s6* gradually increased. The transcriptional expression of *s6* was significantly increased in the LFM+YG2.0 group (*P* < 0.05). The transcriptional expression of *eif4e* was increased in the experimental groups compared with the LFM group and no significantly difference compared to FM group. The transcriptional expression of *4ebp2* in LFM+YG2.0 group was no significant difference compared to FM group (**Figures 2F, G**).

### 3.4 Intestinal morphological analysis

**Figure 3:**
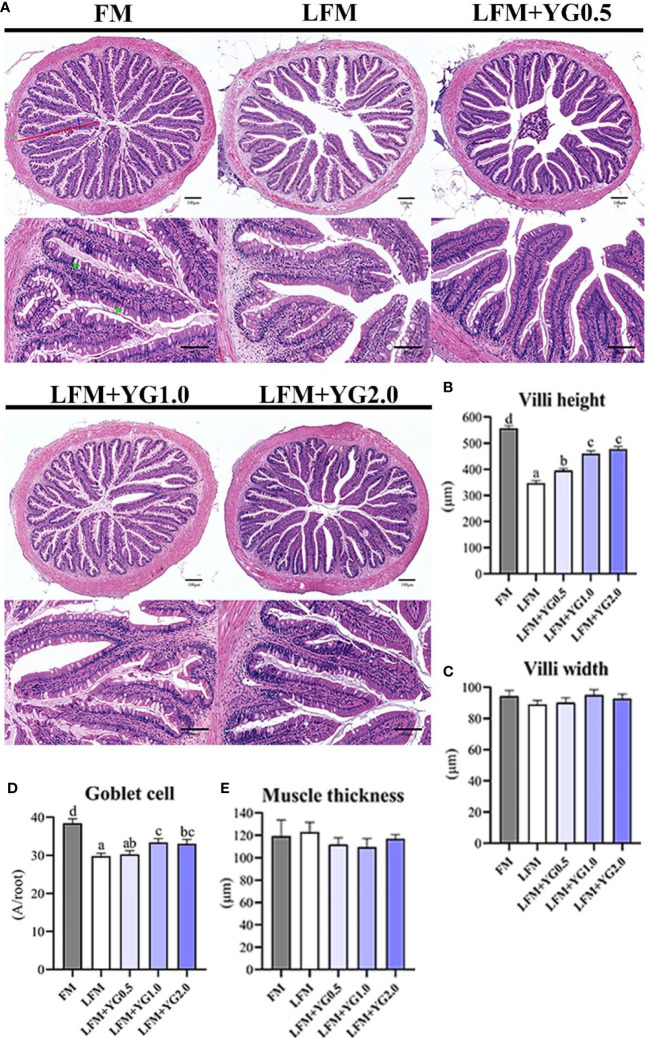
Effects of dietary supplementation with YG on the intestinal morphology of largemouth bass. **(A)** Histological observations of the intestine stained with HE (magnification 100× and 200×). The red line represents the length of villi, the blue line represents the width of villi, the green line represents the thickness of intestinal muscle, and green arrows point to goblet cells. **(B)** Quantitative graph of intestinal villi height. **(C)** Quantitative graph of intestinal villi width. **(D)** Quantitative graph of intestinal goblet cells. **(E)** Quantitative graph of intestinal muscle thickness. Columns represent the mean ± SEM (n = 6). For each index, bars not sharing a common letter indicate significant differences (*P* < 0.05).

Based on the intestinal H&E staining sections, intestinal villi height, villi width, goblet cells and muscular thickness were quantified and are shown in **Figures 3A–E**. With the increase in dietary YG supplementation, the intestinal villi height gradually increased. Dietary YG promoted the intestinal villi height and a significant difference was observed in the experimental group compared with the LFM group (*P* < 0.05) (**Figure 3B**). The number of goblet cells in groups with dietary YG supplementation was higher than that in the LFM group and was significantly increased in the LFM+YG1.0 group (*P* < 0.05). Although the FM treatment had the highest level of goblet cells number (**Figure 3D**). The intestinal villi width and muscular thickness were not significantly affected by dietary YG levels (**Figures 3C, E**).

### 3.5 Intestinal tight junction transcript abundance

**Figure 4:**
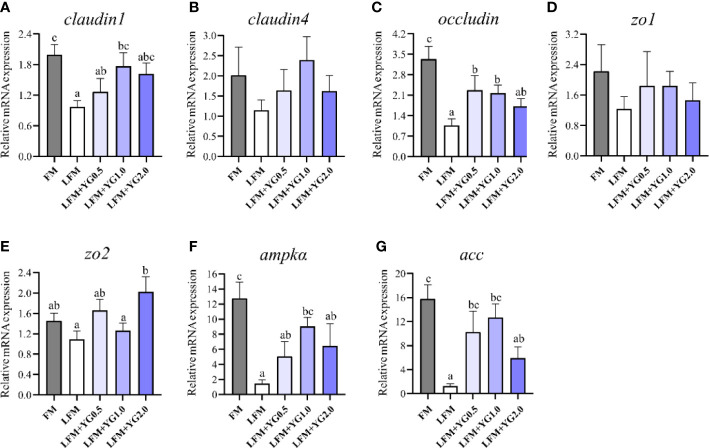
Effects of dietary supplementation with YG on the intestinal tight junction transcript abundance in largemouth bass. **(A)** Relative mRNA expression of *claudin1*. **(B)** Relative mRNA expression of *claudin4*. **(C)** Relative mRNA expression of *occludin*. **(D)** Relative mRNA expression of *zo1*. **(E)** Relative mRNA expression of *zo2*. **(F)** Relative mRNA expression of *ampkα*. **(G)** The Relative mRNA expression of *acc*. Columns represent the mean ± SEM (n = 6). For each index, bars not sharing a common letter indicate significant differences (*P* < 0.05).

The intestinal tight junction transcript abundance analysis is shown in **Figures 4A–E**. With the increase in the dietary YG level, the transcriptional expression of *claudin1* gradually improved, and a significant increase was observed in the LFM+YG1.0 group compared to that in LFM group (*P* < 0.05) (**Figure 4A**). Similar results were found for the transcriptional expression of *occludin* and *zo2*, which was higher in groups with dietary YG supplementation than in the LFM group (**Figures 4C, E**). The expression of *occludin* in the LFM+YG0.5 and LFM+YG1.0 groups was significantly increased compared with that in the LFM group (*P* < 0.05). The expression level of *zo2* in the LFM+YG2.0 treatment was remarkably increased compared with that in the LFM treatment (*P* < 0.05). The transcriptional expression of *claudin4* and *zo1* did not show any significant differences among all groups (**Figures 4B, D**). The relative expression of 5’-AMP-activated protein kinase catalytic subunit α (*ampkα*) and acetyl-CoA carboxylase (*acc*) in middle gut is shown in **Figures 4F, G**. The gene expression levels of *ampkα* and *acc* were remarkably lower in LFM group than the FM group (*P* < 0.05). Nevertheless, their expression levels of them were increased after YG supplementation. The expression level of *ampkα* in the LFM+YG1.0 treatment was remarkably higher than that in the LFM treatment, and the expression level of *acc* was significantly higher in LFM+YG0.5 and LFM+YG1.0 treatments (*P* < 0.05).

### 3.6 Intestinal nonspecific immunity

**Figure 5:**
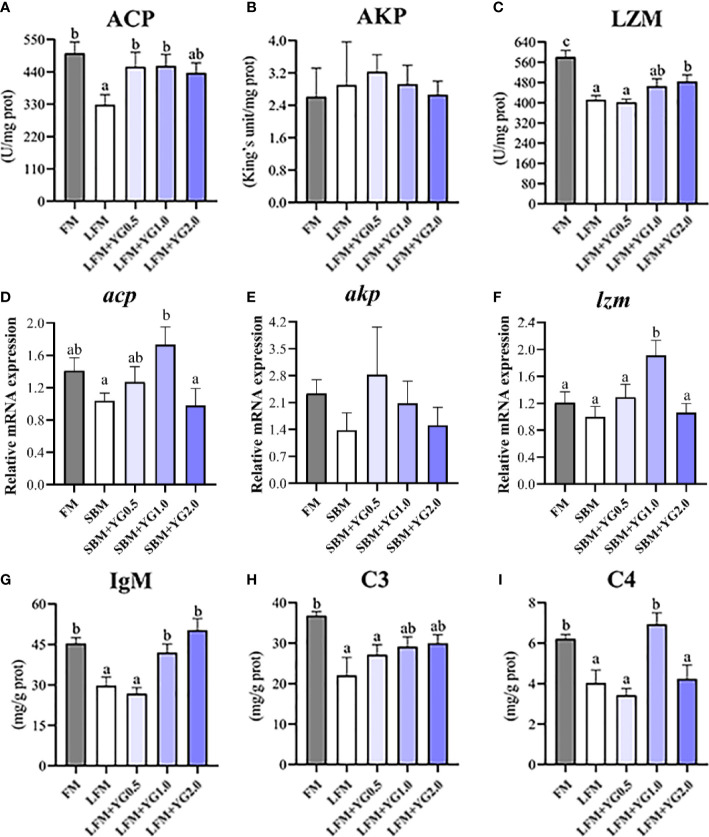
Effects of dietary supplementation with YG on intestinal nonspecific immunity in largemouth bass. **(A)** Acid phosphatase (ACP) (U/mg protein). **(B)** Alkaline phosphatase (AKP) (King’s unit/mg protein). **(C)** Lysozyme (U/mg protein). **(D)** Relative mRNA expression of *acp*. **(E)** Relative mRNA expression of *akp*. **(F)** Relative mRNA expression of *lzm*. **(G)** Immunoglobulin M (IgM) (mg/g protein). **(H)** Complement 3 (C3) (mg/g protein). **(I)** Complement 4 (C4) (mg/g protein). Columns represent the mean ± SEM (n = 6). For each index, bars not sharing a common letter indicate significant differences (*P* < 0.05).

The intestinal nonspecific immunity of the largemouth bass fed dietary YG supplementation is shown in **Figure 5**. With the increase in dietary YG supplementation, the ACP activity gradually increased and it was significantly increased in the LFM+YG0.5 and LFM+YG1.0 groups compared to that in the LFM group (*P* < 0.05). The ACP activity of YG additional treatments was similar with FM group (**Figure 5A**). A similar result was found for LZM. Although the FM treatment had the highest level of LZM activity between the treatments. Dietary YG promoted LZM activity and a significant difference was observed in the LFM+YG2.0 treatment compared to that in LFM group (*P* < 0.05) (**Figure 5C**). The AKP activity was not significantly affected by dietary YG supplementation (**Figure 5B**). Accordingly, the transcriptional expression of *acp* and *lzm* in the experimental groups gradually upregulated and then downregulated with the dietary YG levels, and a remarkable increase was observed in LFM+YG1.0 group compared with that in the LFM group (*P* < 0.05) (**Figures 5D, E**). There was no significant difference in the transcriptional expression of *akp* among all groups (**Figure 5E**). As shown in **Figures 4G-I**, the contents of LGM and C3 in the intestine showed an increasing trend with increased dietary YG levels compared with the LFM group. The LGM content in the LFM+YG1.0 and LFM+YG2.0 groups was significantly increased compared with that in the LFM group (*P* < 0.05) and was not significantly different from that in the FM group. The intestinal C4 content showed an increasing and decreasing trend with increasing dietary YG levels compared with the LFM group. The C4 content in the LFM+YG1.0 group was significantly increased compared with that in the LFM group (*P* < 0.05) and was not significantly different from that in the FM group.

### 3.7 Intestinal antioxidant capacity

**Figure 6:**
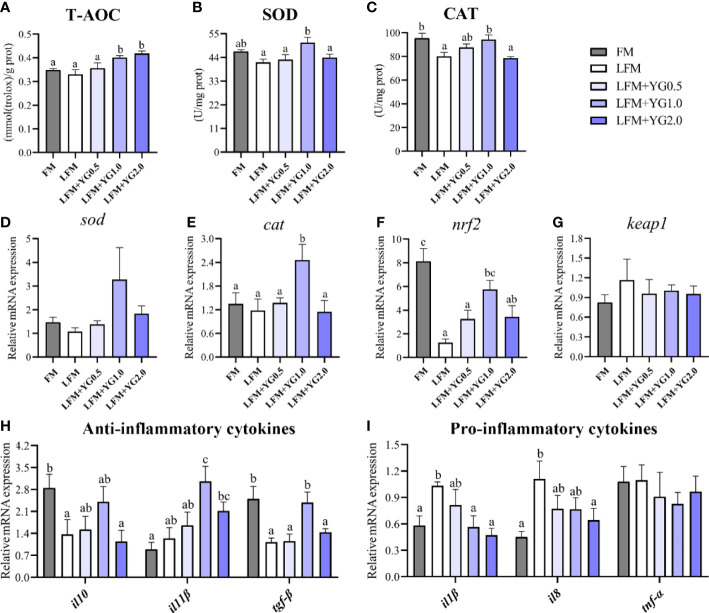
Effects of dietary supplementation with YG on the intestinal antioxidant capacity and inflammatory cytokines in largemouth bass. **(A)** Total antioxidant capacity (T-AOC) (mmol (Trolox)/g prot). **(B)** Superoxide dismutase (SOD) (U/mg prot). **(C)** Catalase (CAT) (U/mg prot). **(D)** Relative mRNA expression of *sod*. **(E)** Relative mRNA expression of *cat*. **(F)** Relative mRNA expression of *nrf2*. **(G)** Relative mRNA expression of *keap1*. **(H)** Relative mRNA expression of anti-inflammatory cytokines (*il10*, *il11β* and *tgf-β*). **(I)** Relative mRNA expression of proinflammatory cytokines (*il1β*, *il8* and *tnf-α*). Columns represent the mean ± SEM (n = 6). For each index, bars not sharing a common letter indicate significant differences (*P* < 0.05).

The intestinal antioxidant parameters are shown in **Figure 6**. The T-AOC of the intestine increased with increasing dietary YG supplementation. The T-AOC in the LFM+YG1.0 and LFM+YG2.0 treatment was significantly increased compared with that in the FM and LFM groups (*P* < 0.05) (**Figure 6A**). The LFM+YG1.0 treatment had the highest level of SOD activity among all groups and was significantly higher than that in the LFM group (*P* < 0.05) (**Figure 6B**). A similar result was observed for CAT, the activity of CAT in the LFM+YG0.5 and LFM+YG1.0 treatments was higher than that in the LFM treatment and was significantly increased in the LFM+YG1.0 treatment compared with the LFM treatment (*P* < 0.05) (**Figure 6C**). As shown in **Figure 6D**, the transcriptional expression of *sod* showed an increasing and decreasing trend with increasing dietary YG levels, although no significant difference was observed among all groups. In addition, with the increase in dietary YG supplementation, the transcriptional expression of *cat* showed an upregulating and then downregulating trend. The highest value was obtained in LFM+YG1.0 group, and it was significantly higher in the LFM+YG1.0 group than that in the FM and LFM groups (*P* < 0.05) (**Figure 6E**). The transcriptional expression of nuclear factor erythroid 2-related factor 2a (*nrf2*) and kelch-like ECH-associated protein 1 (*keap1*) is shown in **Figures 6F, G**. The expression level of *nrf2* in the LFM treatment was remarkably downregulated than that in the FM treatment, meanwhile the expression level of *nrf2* in the LFM+YG1.0 treatment was remarkably upregulated compared with that in the LFM group (*P* < 0.05). There was no significant difference for the expression level of *keap1* among all groups.

### 3.8 Intestinal inflammatory cytokine gene expression

The gene expression levels of inflammatory cytokine in intestinal tissues were determined and are presented in **Figures 6H, I**. As shown in **Figure 6H**, the transcriptional expression of anti-inflammatory cytokines (interleukin 10 (*il-10*), interleukin 11β (*il-11β*) and transforming growth factor β (*tgf-β*)) increased and then decreased with increasing dietary YG supplementation compared with the LFM group, and the highest value appeared in the LFM+YG1.0 group. The expression of *il-10* and *tgf-β* in the LFM+YG1.0 group showed no significant difference with that in FM treatment. The expression of *il-11β* and *tgf-β* in the LFM+YG1.0 group showed a significant difference compared with that in the LFM group (*P* < 0.05). The transcriptional expression of pro-inflammatory cytokines (interleukin 1β (*il1-β*), interleukin 8 (*il-8*) and tumour necrosis factor-α (*tnf-α*)) are shown in **Figure 6I**. Compared with the LFM group, the intestinal expression of *il-1β* and *il-8* showed a decreasing trend with increasing dietary YG levels. Additionally, the downregulation of *il1-β* was significant in the LFM+YG1.0 and LFM+YG2.0 groups, and the downregulation of *il-8* in the LFM+YG2.0 group was significant (*P* < 0.05). However, there was no significant difference between the experimental groups and the FM group. The transcriptional expression of *tnf-α* in all groups showed no significant differences.

## 4 Discussion

An important factor in assessing the suitability of feed additives for farmed animals is the impact on growth performance (23). In this study, we observed that the addition of YG to a diet based on soybean meal as a protein source significantly improved the WGR of largemouth bass, and the WGR was not significantly different from that of the FM group when YG was added at a level of 2.0%. Numerous studies have confirmed that yeast products can be used as additives to improve the growth performance of cultured animals (28–31). Dietary yeast hydrolysate and brewer’s yeast supplementation enhanced the growth performance of Pacific white shrimp (32). Growth improvement was observed with dietary probiotic yeast supplementation in Gangetic mystus (*Mystus cavasius*) (15). However, this study found that the FE and FR of largemouth bass were not increased with YG supplementation. Therefore, dietary YG may improve growth through endogenous factors. The TOR signaling pathway plays an important role in sensing nutrients and regulating the growth of the organism by coordinating anabolism and catabolism (33). Moreover, a positive correlation was observed between the growth and phosphorylation activation of the TOR pathway in cultured animals (34). A previous study reported that the addition of guanidinoacetic acid promoted muscle cell growth by upregulating the TOR signaling pathway (35). Dietary Clostridium butyricum CBG01 enhanced the growth performance of Pacific white shrimp, and the TOR signaling pathway was shown to be activated (36). Our study also observed that dietary YG significantly increased the transcriptional and protein levels of *tor* and *s6*. YG contains some active substances, such as mannitol, that may be essential for the activation of the TOR signaling pathway, and this needs to be further validated. Therefore, the possible mechanism by which growth was improved by YG may be partly due to the activation of the TOR signaling pathway.

The intestinal physical barrier is important in maintaining intestinal health and resisting the invasion of pathogenic bacteria (37). The intestinal villi height, width and muscle thickness are the direct indicators of the morphological structure and functional integrity of the intestinal mucosa (38). In this study, the values of villi height significantly increased after largemouth bass were fed the diet with YG supplementation. In addition, the number of goblet cells can reflect to some extent the local immune status of the gut (39). This study found that YG increased the number of intestinal goblet cells when experimental fish were fed a diet containing soybean meal, which was consistent with the results for innate immunity mentioned above. These results suggested that YG enhanced the intestinal physical barrier effect, which may be related to improvement in the integrity of the gut and promotion of the differentiation of intestinal cells. Tight junction proteins, consisting of transmembrane proteins (claudins and occludin) and cytoplasmic proteins (zos), are an important component of the physical barrier of the intestine (40). This research showed that the addition of YG to the low fishmeal diet markedly increased the expression of *claudin1*, *occludin* and *zo2* in the intestine, indicating that YG can alleviate the damage to the intestinal physical barrier caused by a diet containing soybean meal. Glucan is as important a component of YG as MOS. Our results are consistent with previous studies that reported that glucan and MOS attenuated barrier dysfunction and enhanced gut health in broiler chickens (41). Nevertheless, the possible mechanism by which yeast products regulate the intestinal physical barrier is unknown. Accumulating evidence has shown that the AMPK signaling pathway can further activate the barrier function of intestinal epithelial cells by enhancing the interaction between claudin and occludin (42). This study showed that dietary YG upregulated the expression levels of *ampk* and *acc* in the gut. Another previous study reported that AMPK inhibition due to metabolic disorders coexists with impaired epithelial barrier function (43). Glutmate has been reported to activate AMPK pathway to enhance intestinal barrier function by promoting the assembly of tight junctions (44). Therefore, we speculate that YG may regulate the AMPK signaling pathway to improve the intestinal physical barrier, but the specific mechanism requires subsequent investigation.

In fish, the innate immune system consists of ACP, AKP and LZM. It is the first line of intestinal immune defense and plays a vital role in protecting aquatic animals against invading microorganisms, such as pathogenic bacteria (45). In this study, the intestinal ACP and LZM activities were significantly decreased by soybean meal. However, YG supplementation enhanced the activities of ACP and LZM, and the activities of ACP and LZM in the LFM+YG1.0 group were not different from those in the FM group. This was confirmed by the results of gene expression analysis, where YG upregulated the expression levels of *acp* and *lzm*. Similar results were found in a previous study in which the dietary live yeast *Hanseniaspora opuntiae* C21 in juvenile sea cucumber (*Apostichopus japonicus*) and the dietary protease-complex in shrimp *(L. vannamei.*) increased ACP activity (46, 47). Immunoglobulins (IgM) and complements 3 and 4 (C3 and C4) are important components of the innate immune system of fish and play a positive role in preventing the invasion of external microorganisms (48). In the present study, the intestinal IgM and C4 contents were significantly increased in fish fed with dietary YG supplementation compared to those in the LFM group. Consistent with this study, the immunity-promoting effects of yeast products have been reported previously (23, 49). However, previous results have shown that yeast products can increase innate immunity levels in organs, such as the kidneys and liver, and the present study demonstrated that yeast products could also improve the intestinal immune capacity of aquatic animals. However, there is no precise explanation of how dietary YG works to increase the intestinal immunoreactive substance content. It is speculated that YG may activate the complement system to secrete C4 and promote the secretion of IgM in intestine. The exact mechanism needs to be further explored.

The antioxidant system is able to scavenge reactive oxygen species and alleviate damage to cells; thus, antioxidant capacity can be used to reflect the health status of the body (50). The present results showed that dietary YG supplementation significantly increased T-AOC in the intestine. SOD and CAT are important enzymes in the antioxidant defense system. SOD reduces reactive oxygen species to hydrogen peroxide, and CAT decomposes hydrogen peroxide into water and oxygen (51). This study found that the contents of SOD and CAT were increased when fish were fed the diet supplemented with YG. Accordingly, the mRNA expression levels of *sod* and *cat* were also increased in the YG supplementation groups compared with the LFM group. Our results are consistent with a previous study showing that YG improved antioxidant capacity in broiler chickens (52). MOS is an important component in YG and was reported to improve oxidative status and ameliorate heat stress-induced intestinal damage in animals (53, 54). Therefore, dietary YG may act through MOS to help protect the intestine from oxidative stress. Nuclear factor-erythroid factor 2-related factor 2 (NRF2) is a transcription factor that regulates the expression of antioxidant genes (48, 55). The transcriptional expression of *nrf2* in this study was increased and the transcriptional expression of *keap1* was decreased in the YG-supplemented groups, indicating that the improvement in antioxidant capacity might have resulted from the activation of the Nrf2/Keap1 pathway. As a feed additive, YG can increase the antioxidant capacity of livestock and poultry animals. The present study further found that YG was effective in increasing the antioxidant capacity of the aquatic animal intestine through the Nrf2/Keap1 signaling pathway.

Cytokines trigger a disease-resistance response of the immune system and help to enhance the immune response in the body (21). Anti-inflammatory cytokines (such as IL-10, IL-11β and TGF-β) and proinflammatory cytokines (such as IL-1β, IL-8 and TNF-α) are usually detected to assess the immune response (56). Live yeast have been confirmed to reduce the expression of proinflammatory cytokines and increase the expression of anti-inflammatory cytokines in porcine intestinal epithelial cells following bacterial invasion (57). As an extract of yeast cell walls, YG was found to have similar effects in this study. Dietary YG upregulated the mRNA expression of *il-10*, *il-11β* and *tgf-β* but downregulated the mRNA expression of *il-1β* and *il-8*. MOS and glucan are essential components of both live yeast and YG and have been shown to have therapeutic effects on inflammation (16, 58). In addition, the current study showed that dietary YG can activate the Nrf2/Keap1 signaling pathway. Previous studies have reported that the expression of inflammatory cytokines is also regulated by the Nrf2/Keap1 signaling pathway (59). Therefore, the possible mechanisms underlying the alterations in intestinal inflammatory factors in this study may be partially attributable to regulation of the Nrf2/Keap1 signaling pathway by YG. In summary, dietary YG can regulate intestinal antioxidant capacity and inflammatory cytokines through the Nrf2/Keap1 signaling pathway in largemouth bass.

## 5 Conclusions

The current study explored the impacts of YG as a feed additive for aquatic animals. The results reveal that a low fishmeal diet had negative impacts on growth performance and intestinal health and decreased the survival rate after bacterial challenge of largemouth bass. However, dietary YG supplementation (0.5-2%) could improve the growth performance, intestinal health and survival rate by optimizing the gut morphology and enhancing the intestinal antioxidant capacity and nonspecific immunity. Furthermore, dietary YG improves growth performance and intestinal health by regulating through the TOR, AMPK and Nrf2/Keap1 pathways. Therefore, our findings can provide a new strategy for the replacement of fishmeal by plant proteins in aquaculture and provide a reference for the application of YG diets in aquatic animals.

## Data availability statement

The original contributions presented in the study are included in the article/supplementary materials. Further inquiries can be directed to the corresponding author.

## Ethics statement

The animal study was reviewed and approved by Institute of Hydrobiology, Chinese Academy of Sciences.

## Author contributions

WC and HL made major contributions to this work; The main contributions of WC were the analysis of data and drafting of the manuscript, and HL reviewed and corrected the manuscript. SX, JD, XZ, YY, DH, JJ, LF, JY, FY, LHH and LYH participated in the experimental design and sample collection. All authors contributed to the article and approved the submitted version.

## References

[1] Takakuwa F Murashita K Noguchi Y Inui T Watanabe K Sugiyama S. Effects of long-term feeding of fishmeal-free diet on growth parameters, bile acid status, and bile acid-related gene expression of yearling red sea bream *Pagrus major* (Temminck & schlegel, 1843). Aquaculture (2023) 570:739444. doi: 10.1016/j.aquaculture.2023.739444

[2] Li S Dai M Qiu H Chen N. Effects of fishmeal replacement with composite mixture of shrimp hydrolysate and plant proteins on growth performance, feed utilization, and target of rapamycin pathway in largemouth bass, *Micropterus salmoides* . Aquaculture (2021) 533:736185. doi: 10.1016/j.aquaculture.2020.736185

[3] Yang X He Y Chi S Tan B Lin S Dong X. Supplementation with saccharomyces cerevisiae hydrolysate in a complex plant protein, low-fishmeal diet improves intestinal morphology, immune function and vibrio harveyi disease resistance in *Epinephelus coioides* . Aquaculture (2020) 529:735655. doi: 10.1016/j.aquaculture.2020.735655

[4] Liu Y Chang H Lv W Ma S Qiu G Lu S. Physiological response of rainbow trout (*Oncorhynchus mykiss*) to graded levels of novel *Chlorella sorokiniana* meal as a single fishmeal alternative or combined with black soldier fly larval meal. Aquaculture (2022) 561:738715. doi: 10.1016/j.aquaculture.2022.738715

[5] Xie S Liu Y Zeng S Niu J. Tian l. partial replacement of fish-meal by soy protein concentrate and soybean meal based protein blend for juvenile pacific white shrimp, *Litopenaeus vannamei* . Aquaculture (2016) 464:296–302. doi: 10.1016/j.aquaculture.2016.07.002

[6] Biswas A Takakuwa F Yamada S Matsuda A Saville RM LeBlanc A. Methanotroph (*Methylococcus capsulatus*, bath) bacteria meal as an alternative protein source for Japanese yellowtail, *Seriola quinqueradiata* . Aquaculture (2020) 529:735700. doi: 10.1016/j.aquaculture.2020.735700

[7] Lu Q Xi L Liu Y Gong Y Su J Han D. Effects of dietary inclusion of *Clostridium autoethanogenum* protein on the growth performance and liver health of largemouth bass (*Micropterus salmoides*). Front Mar Sci (2021) 8:764964. doi: 10.3389/fmars.2021.764964

[8] Su J Gong Y Cao S Lu F Han D Liu H. Effects of dietary tenebrio molitor meal on the growth performance, immune response and disease resistance of yellow catfish (*Pel teobagrus fulvidraco*). Fish Shellfish Immunol (2017) 69:59–66. doi: 10.1016/j.fsi.2017.08.008 28807649

[9] Hu Y Zhang J Xue J Chu W Hu Y. Effects of dietary soy isoflavone and soy saponin on growth performance, intestinal structure, intestinal immunity and gut microbiota community on rice field eel (*Monopterus albus*). Aquaculture (2021) 537:736506. doi: 10.1016/j.aquaculture.2021.736506

[10] Ahmed M Liang H Chisomo Kasiya H Ji K Ge X Ren M. Complete replacement of fish meal by plant protein ingredients with dietary essential amino acids supplementation for juvenile blunt snout bream (*Megalobrama amblycephala*). Aquacult Nutr (2019) 25:205–14. doi: 10.1111/anu.12844

[11] Ma R Meng Y Zhang W Mai K. Comparative study on the organoleptic quality of wild and farmed large yellow croaker *Larimichthys crocea* . J Oceanol Limnol (2020) 38:260–74. doi: 10.1007/s00343-019-8353-0

[12] Gu M Jia Q Zhang Z Bai N Xu X Xu B. Soya-saponins induce intestinal inflammation and barrier dysfunction in juvenile turbot (Scophthalmus maximus). Fish Shellfish Immunol (2018) 77:264–72. doi: 10.1016/j.fsi.2018.04.004 29625242

[13] Lin J Liao Y Li X Lu K Song K Wang L. Effects of dietary creatine levels on the growth, muscle energy metabolism and meat quality of spotted seabass (*Lateolabrax maculatus*) fed low-fishmeal diets. Aquaculture (2023) 565:739075. doi: 10.1016/j.aquaculture.2022.739075

[14] To V Liou C. Taurine supplementation enhances the replacement level of fishmeal by soybean concentrate in diets of juvenile pacific white shrimp (*Litopenaeus vannamei* Boone, 1931). Aquacult Res (2021) 52:3771–84. doi: 10.1111/are.15222

[15] Banu MR Akter S Islam MR Mondol MN Hossain MA. Probiotic yeast enhanced growth performance and disease resistance in freshwater catfish gulsa tengra, *Mystus cavasius* . Aquacult Rep (2020) 16:100237. doi: 10.1016/j.aqrep.2019.100237

[16] Neamat-Allah ANF Abd El Hakim Y Mahmoud EA. Alleviating effects of β-glucan in *Oreochromis niloticus* on growth performance, immune reactions, antioxidant, transcriptomics disorders and resistance to *Aeromonas sobria* caused by atrazine. Aquacult Res (2020) 51:1801–12. doi: 10.1111/are.14529

[17] Rungrassamee W Kingcha Y Srimarut Y Maibunkaew S Karoonuthaisiri N Visessanguan W. Mannooligosaccharides from copra meal improves survival of the pacific white shrimp (*Litopenaeus vannamei*) after exposure to *Vibrio harveyi* . Aquaculture (2014) 434:403–10. doi: 10.1016/j.aquaculture.2014.08.032

[18] Qin L Ji W Wang J Li B Hu J Wu X. Effects of dietary supplementation with yeast glycoprotein on growth performance, intestinal mucosal morphology, immune response and colonic microbiota in weaned piglets. Food Funct (2019) 10:2359–71. doi: 10.1039/C8FO02327A 30972390

[19] Li Y Yang Y Ji Q Song J Wang L Liu B. The function of *Apostichopus japonicas* catalase in sea cucumber intestinal immunity. Aquaculture (2020) 521:735103. doi: 10.1016/j.aquaculture.2020.735103

[20] Yan L Yang C Tang J. Disruption of the intestinal mucosal barrier in *Candida albicans* infections. Microbiol Res (2013) 168:389–95. doi: 10.1016/j.micres.2013.02.008 23545353

[21] Feng J Cai Z Chen Y Zhu H Chang X Wang X. Effects of an exopolysaccharide from *Lactococcus lactis* z-2 on innate immune response, antioxidant activity, and disease resistance against *Aeromonas hydrophila* in *Cyprinus carpio* l. Fish Shellfish Immunol (2020) 98:324–33. doi: 10.1016/j.fsi.2020.01.037 31981775

[22] Jiang W Deng Y Liu Y Qu B Jiang J Kuang S. Dietary leucine regulates the intestinal immune status, immune-related signalling molecules and tight junction transcript abundance in grass carp (*Ctenopharyngodon idella*). Aquaculture (2015) 444:134–42. doi: 10.1016/j.aquaculture.2015.04.005

[23] Gong Y Yang F Hu J Liu C Liu H Han D. Effects of dietary yeast hydrolysate on the growth, antioxidant response, immune response and disease resistance of largemouth bass (*Micropterus salmoides*). Fish Shellfish Immunol (2019) 94:548–57. doi: 10.1016/j.fsi.2019.09.044 31539573

[24] AOAC. Official methods of analysis of AOAC international. eighteenth ed. Arlington, Virginia, USA: Association of Official Analytical Chemists (2005).

[25] Cai W Liu H Fu L Han D Zhu X Jin J. Dietary inosine monophosphate improved liver health and flesh quality of gibel carp (*Carassius auratus gibelio*) *via* activating AMPK signalling pathway and enhancing the contents of muscle fat and flavour substance. Front Mar Sci (2022) 9:940732. doi: 10.3389/fmars.2022.940732

[26] Yu H Zhang L Chen P Liang X Cao A Han J. Dietary bile acids enhance growth, and alleviate hepatic fibrosis induced by a high starch diet via AKT/FOXO1 and cAMP/AMPK/SREBP1 pathway in Micropterus salmoides. Front Physiol (2019) 10:1430. doi: 10.3389/fphys.2019.01430 31824338 PMC6882294

[27] Vandesompele J Preter KD Roy NV Paepe AD. Accurate normalization of real-time quantitative RT-PCR data by geometric averaging of multiple internal control genes. Genome Biol (2002) 3:12. doi: 10.1186/gb-2002-3-7-research0034 PMC126239 12184808

[28] Zhang P Fu L Liu H Huda N-U Zhu X Han D. Effects of inosine 5′-monophosphate supplementation in high fishmeal and high soybean diets on growth, immune-related gene expression in gibel carp (*Carassius auratus gibelio* var. CAS III), and its challenge against *Aeromonas hydrophila* infection. Fish Shellfish Immunol (2019) 86:913–21. doi: 10.1016/j.fsi.2018.12.016 30550991

[29] Jahan N Islam SMM Rohani MF Hossain MT Shahjahan M. Probiotic yeast enhances growth performance of rohu (*Labeo rohita*) through upgrading hematology, and intestinal microbiota and morphology. Aquaculture (2021) 545:737243. doi: 10.1016/j.aquaculture.2021.737243

[30] Fu Y Guo J Wu Z Yu X Guo Y Huang D. Effects of dietary chromium yeast levels on growth performance, anti-oxidative capacity, immune response and flesh quality of abalone *Haliotis discus hannai* . Aquaculture (2022) 557:738291. doi: 10.1016/j.aquaculture.2022.738291

[31] Richard N Costas B Machado M Fernández-Boo S Girons A Dias J. Inclusion of a protein-rich yeast fraction in rainbow trout plant-based diet: consequences on growth performances, flesh fatty acid profile and health-related parameters. Aquaculture (2021) 544:737132. doi: 10.1016/j.aquaculture.2021.737132

[32] Jin M Xiong J Zhou Q-C Yuan Y Wang X-X Sun P. Dietary yeast hydrolysate and brewer’s yeast supplementation could enhance growth performance, innate immunity capacity and ammonia nitrogen stress resistance ability of pacific white shrimp (*Litopenaeus vannamei*). Fish Shellfish Immunol (2018) 82:121–9. doi: 10.1016/j.fsi.2018.08.020 30099143

[33] Jiang H Bian F Zhou H Wang X Wang K Mai K. Nutrient sensing and metabolic changes after methionine deprivation in primary muscle cells of turbot (*Scophthalmus maximus* l.). J Nutr Biochem (2017) 50:74–82. doi: 10.1016/j.jnutbio.2017.08.015 29040838

[34] Liu Y Lu Q Xi L Gong Y Su J Han D. Effects of replacement of dietary fishmeal by cottonseed protein concentrate on growth performance, liver health, and intestinal histology of largemouth bass (*Micropterus salmoides*). Front Physiol (2021) 12:764987. doi: 10.3389/fphys.2021.764987 34992547 PMC8724133

[35] Wang Y Ma J Qiu W Zhang J Feng S Zhou X. Guanidinoacetic acid regulates myogenic differentiation and muscle growth through miR-133a-3p and miR-1a-3p Co-mediated Akt/mTOR/S6K signaling pathway. Int J Mol Sci (2018) 19:2837. doi: 10.3390/ijms19092837 30235878 PMC6163908

[36] Luo K Tian X Wang B Wei C Wang L Zhang S. Evaluation of paraprobiotic applicability of *Clostridium butyricum* CBG01 in improving the growth performance, immune responses and disease resistance in pacific white shrimp, *Penaeus vannamei* . Aquaculture (2021) 544:737041. doi: 10.1016/j.aquaculture.2021.737041

[37] Shi Y Zhong L Zhong H Zhang J Che C Fu G. Taurine supplements in high-fat diets improve survival of juvenile *Monopterus albus* by reducing lipid deposition and intestinal damage. Aquaculture (2022) 547:737431. doi: 10.1016/j.aquaculture.2021.737431

[38] Wei L Wu P Zhou X Jiang W Liu Y Kuang S. Dietary silymarin supplementation enhanced growth performance and improved intestinal apical junctional complex on juvenile grass carp (*Ctenopharyngodon idella*). Aquaculture (2020) 525:735311. doi: 10.1016/j.aquaculture.2020.735311

[39] Liu Y Zhong L Chen T Shi Y Hu Y Zeng J. Dietary sanguinarine supplementation on the growth performance, immunity and intestinal health of grass carp (*Ctenopharyngodon idellus*) fed cottonseed and rapeseed meal diets. Aquaculture (2020) 528:735521. doi: 10.1016/j.aquaculture.2020.735521

[40] Chasiotis H Kolosov D Bui P Kelly SP. Tight junctions, tight junction proteins and paracellular permeability across the gill epithelium of fishes: a review. Respir Physiol Neurobiol (2012) 184:269–81. doi: 10.1016/j.resp.2012.05.020 22640933

[41] Anwar MI Muhammad F Awais MM Akhtar M. A review of β-glucans as a growth promoter and antibiotic alternative against enteric pathogens in poultry. World’s Poult Sci J (2017) 73:651–61. doi: 10.1017/S0043933917000241

[42] Zhu M Sun X Du M. AMPK in regulation of apical junctions and barrier function of intestinal epithelium. Tissue Barriers (2018) 6:1–13. doi: 10.1080/21688370.2018.1487249 PMC6179128 30130441

[43] Meddings J. The significance of the gut barrier in disease. Gut (2007) 57:438–40. doi: 10.1136/gut.2007.143172 18334657

[44] Wang B Wu Z Ji Y Sun K Dai Z Wu G. L-glutamine enhances tight junction integrity by activating CaMK kinase 2–AMP-Activated protein kinase signaling in intestinal porcine epithelial cells. J Nutr (2016) 146:501–8. doi: 10.3945/jn.115.224857 26865645

[45] Ye G. Low-gossypol cottonseed protein concentrate used as a replacement of fish meal for juvenile hybrid grouper (*Epinephelus fuscoguttatus* ♀ × *Epinephelus lanceolatus* ♂): effects on growth performance, immune responses and intestinal microbiota. Aquaculture (2020) 524:735309. doi: 10.1016/j.aquaculture.2020.735309 31704202

[46] Ma Y Liu Z Yang Z Li M Liu J Song J. Effects of dietary live yeast *Hanseniaspora opuntiae* C21 on the immune and disease resistance against *Vibrio splendidus* infection in juvenile sea cucumber *Apostichopus japonicus* . Fish Shellfish Immunol (2013) 34:66–73. doi: 10.1016/j.fsi.2012.10.005 23063538

[47] Song H Tan B Chi S Liu Y Chowdhury MAK Dong X. The effects of a dietary protease-complex on performance, digestive and immune enzyme activity, and disease resistance of *Litopenaeus vannamei* fed high plant protein diets. Aquacult Res (2017) 48:2550–60. doi: 10.1111/are.13091

[48] Yu Z Zhao L Zhao J-L Xu W Guo Z Zhang A-Z. Dietary *Taraxacum mongolicum* polysaccharide ameliorates the growth, immune response, and antioxidant status in association with NF-κB, Nrf2 and TOR in jian carp (*Cyprinus carpio* var. jian). Aquaculture (2022) 547:737522. doi: 10.1016/j.aquaculture.2021.737522

[49] Yuan X Liu W Liang C Sun C Xue Y Wan Z. Effects of partial replacement of fish meal by yeast hydrolysate on complement system and stress resistance in juvenile jian carp (*Cyprinus carpio* var. jian). Fish Shellfish Immunol (2017) 67:312–21. doi: 10.1016/j.fsi.2017.06.028 28606860

[50] Wu P Yang W Dong Y Wang Y Zhang Y Zou X. Feasibility of cultivation of *Spinibarbus sinensis* with coconut oil and its effect on disease resistance (nonspecific immunity, antioxidation and mTOR and NF-kB signaling pathways). Fish Shellfish Immunol (2019) 93:726–31. doi: 10.1016/j.fsi.2019.06.052 31265912

[51] Shi B Yuan Y Jin M Betancor MB Tocher DR Jiao L. Transcriptomic and physiological analyses of hepatopancreas reveal the key metabolic changes in response to dietary copper level in pacific white shrimp *Litopenaeus vannamei* . Aquaculture (2021) 532:736060. doi: 10.1016/j.aquaculture.2020.736060

[52] Wassie T Cheng B Zhou T Gao L Lu Z Wang J. *Enteromorpha* polysaccharide and yeast glycoprotein mixture improves growth, antioxidant activity, serum lipid profile and regulates lipid metabolism in broiler chickens. Poult Sci (2022) 101:102064. doi: 10.1016/j.psj.2022.102064 36055019 PMC9445391

[53] Cheng Y Chen Y Chen R Su Y Zhang R He Q. Dietary mannan oligosaccharide ameliorates cyclic heat stress-induced damages on intestinal oxidative status and barrier integrity of broilers. Poult Sci (2019) 98:4767–76. doi: 10.3382/ps/pez192 31005999

[54] Hutsko SL Meizlisch K Wick M Lilburn MS. Early intestinal development and mucin transcription in the young poult with probiotic and mannan oligosaccharide prebiotic supplementation. Poult Sci (2016) 95:1173–8. doi: 10.3382/ps/pew019 26944966

[55] Rajput S Zhang C Feng Y Wei X Khalil M Rajput I. Proanthocyanidins alleviates AflatoxinB1-induced oxidative stress and apoptosis through mitochondrial pathway in the bursa of fabricius of broilers. Toxins (2019) 11:157. doi: 10.3390/toxins11030157 30857375 PMC6468869

[56] Tie H Jiang W Feng L Wu P Liu Y Kuang S. Dietary nucleotides in the diets of on-growing grass carp (*Ctenopharyngodon idella*) suppress *Aeromonas hydrophila* induced intestinal inflammation and enhance intestinal disease-resistance *via* NF-κB and TOR signaling. Aquaculture (2021) 533:736075. doi: 10.1016/j.aquaculture.2020.736075

[57] Wang W Li Z Han Q Guo Y Zhang B D’inca R. Dietary live yeast and mannan-oligosaccharide supplementation attenuate intestinal inflammation and barrier dysfunction induced by *Escherichia coli* in broilers. Br J Nutr (2016) 116:1878–88. doi: 10.1017/S0007114516004116 27989252

[58] Neamat-Allah ANF El-Murr A elhakeem I Abd El-Hakim Y. Dietary supplementation with low molecular weight sodium alginate improves growth, haematology, immune reactions and resistance against *Aeromonas hydrophila* in *Clarias gariepinus* . Aquacult Res (2019) 50:1547–56. doi: 10.1111/are.14031

[59] Wu L Xu W Li H Dong B Geng H Jin J. Vitamin c attenuates oxidative stress, inflammation, and apoptosis induced by acute hypoxia through the Nrf2/Keap1 signaling pathway in gibel carp (*Carassius gibelio*). Antioxidants (2022) 11:935. doi: 10.3390/antiox11050935 35624798 PMC9137936

